# The complete mitochondrial genome of *Aglaia odorata*, insights into its genomic structure and RNA editing sites

**DOI:** 10.3389/fpls.2024.1362045

**Published:** 2024-03-06

**Authors:** Zhigang Hao, Zhiping Zhang, Jinan Zhang, Xiufen Cui, Jianqiang Li, Laixin Luo, Yingbin Li

**Affiliations:** ^1^ Department of Pesticide Science, State Key Laboratory for Conservation and Utilization of Bio-Resource in Yunnan, College of Plant Protection, Yunnan Agricultural University, Kunming, Yunnan, China; ^2^ Sanya Institute of China Agricultural University, Sanya, Hainan, China; ^3^ Hainan Seed Industry Laboratory, Sanya, Hainan, China; ^4^ Department of Plant Pathology, Beijing Key Laboratory of Seed Disease Testing and Control, China Agricultural University, Beijing, China; ^5^ MOA Key Lab of Pest Monitoring and Green Management, China Agricultural University, Beijing, China

**Keywords:** *Aglaia odorata*, mitochondrial genome, recombination, MTPT, RNA editing

## Abstract

*Aglaia odorata*, native to Guangdong, Guangxi, and Hainan provinces in China, has long been utilized as an herbal remedy in ancient China. In this study, we assembled and annotated the complete mitochondrial genome (mitogenome) of *A. odorata*, which spans a total length of 537,321 bp. Conformation of the *A. odorata* recombination was verified through PCR experiments and Sanger sequencing. We identified and annotated 35 protein-coding genes (PCGs), 22 tRNA genes, and 3 rRNA genes within the mitogenome. Analysis of repeated elements revealed the presence of 192 SSRs, 29 pairs of tandem repeats, and 333 pairs of dispersed repeats in the *A. odorata* mitogenome. Additionally, we analyzed codon usage and mitochondrial plastid DNAs (MTPTs). Twelve MTPTs between the plastome and mitogenome of *A. odorata* were identified, with a combined length of 2,501 bp, accounting for 0.47% of the mitogenome. Furthermore, 359 high-confidence C to U RNA editing sites were predicted on PCGs, and four selected RNA editing sites were specially examined to verify the creation of start and/or stop codons. Extensive genomic rearrangement was observed between *A. odorata* and related mitogenomes. Phylogenetic analysis based on mitochondrial PCGs were conducted to elucidate the evolutionary relationships between *A. odorata* and other angiosperms.

## Introduction

*Aglaia odorata* Lour. belongs to the *Aglaia* genus within the Meliaceae family. It is native to Guangdong, Guangxi and Hainan provinces in China, as well as various Southeast Asian countries (Zhang et al., 2020b). This plant is commonly found in sparsely forested areas and shrubbery within low-altitude mountain regions (Liu et al., 2014). In ancient China, *A. odorata* has traditionally been employed as an herbal remedy for treating heart diseases, bruises, traumatic injuries, and fever (Yang et al., 2022). Modern pharmacological studies indicate that *A. odorata* exhibits anti-cancer, anti-inflammatory, antibacterial, and antiviral activities (Inad et al., 2001). Despite its economic value, there has been limited research on it, and the genomic information of this species is still lacking at present. So far, in various databases such as the GenBank database, only the chloroplast genome resource is available (accession number: NC_048994.1).

Mitochondria play a pivotal role in synthesizing and converting energy for diverse cellular physiological processes, rendering them essential for plant growth and development (Ye et al., 2017). They transform biomass energy into chemical energy via phosphorylation and participate in cellular processes such as cell division, differentiation, and apoptosis (Kroemer and Reed, 2000; van Loo et al., 2002; Bonora et al., 2014). Mitochondria stand as distinctive cellular structures separate from the nucleus, housing their own genome. This genetic material is inherited in a haploid, asexual, and maternal fashion (Cheng et al., 2021). Following the endosymbiotic theory, mitochondria’s origins trace back to the mutualistic relationship between alpha-bacteria and archaea-derived host cells, eventually evolved into integral organelles within eukaryotic cells (Roger et al., 2017). While mitogenomes, like to plastidial genomes, are maternally inherited and encompass a smaller gene set, significant evolutionary distinctions exist between these two genomes. Compared to the mitogenomes, the plastidial genomes are relatively compact and remarkably conserved. Plant mitogenomes display significant size variation, spanning from 60 kb to over 11 Mb among different species, a considerably broader range than what is observed in plastid genomes (Sloan et al., 2012; Skippington et al., 2015). Higher plant mitogenomes exhibit linear, circular, complex branching and reticular structures, whereas the majority of plant plastidial genomes have a circular structure (Cheng et al., 2017; Kozik et al., 2019; Fischer et al., 2022). Plant mitogenomes tend to exhibit higher mutation rates in comparison to nuclear genomes, a consequence attributed to the absence of robust DNA repair systems (Gualberto et al., 2014; Morley et al., 2019). This higher mutation rate contributes to rearrangements, duplications, and the generation of subgenomic configurations within the mitogenome. Furthermore, certain plant mitogenomes have assimilated genes through horizontal gene transfer from external organisms. This occurrence is especially prevalent in higher plants, where they have incorporated several plastid sequences from neighboring chloroplasts. This evolutionary process has transpired over an extended timeframe and is likely ongoing (Choi and Park, 2021; Garcia et al., 2021; Lin et al., 2022). Currently, the availability of mitogenome resources for the plants is limited.

In this study, we assembled and annotated the mitogenome and plastidial genome of *A. odorata*, analyzed the codon usage, repeated elements, and mitochondrial plastid DNAs (MTPTs). We also analyzed the RNA editing sites in mitochondrial PCGs. Lastly, we inferred the phylogenetic relationships of *A. odorata* and other angiosperms based on mitochondrial PCGs. As the first reported mitogenome within genus Aglaia, this study provides valuable reference for mitogenome analysis in Aglaia species. Additionally, it offers important insights into RNA editing, mitochondrial genome evolution, genome rearrangement, and phylogenetics of angiosperms. Furthermore, we also provide reliable genomic resources for studying the organelle genomes of Meliaceae plants.

## Materials and methods

### Plant sampling, DNA extracting and sequencing

The fresh leaves of *A. odorata* were collected in Sanya, Hainan, China. These specimens have been deposited in our lab (Seed Health Centre of China Agricultural University, Sanya Institute of China Agricultural University and Yunnan Agricultural University). Genomic DNA was extracted using the Tiangen Biotech DNA kit (Beijing). For library construction, we utilized the NEBNext^®^ library building kit with an insert size of 350 bp. The constructed DNA library was sequenced on the NovaSeq 6000 platform at Benagen (Wuhan, China). To ensure data quality, we applied Trimmomatic (Bolger et al., 2014) to remove low-quality sequences, including those with a quality value (Q) of less than or equal to 5, which accounted for more than 50% of the total bases, as well as sequences containing more than 10% “N” bases. Furthermore, the plant sample used for Illumina sequencing was also subjected to Oxford Nanopore sequencing based on PromethION devices. Purified DNA was prepared for long-read sequencing following the protocol outlined in the SQK-LSK109 genomic sequencing kit (ONT, Oxford, UK).

### RNA extracting and sequencing

For long non-coding RNA (lncRNA) extraction, total RNA was isolated from fresh *A*. *odorata* leaves using a high-quality RNA extraction kit (TRIzol^®^ Reagent, Thermo Fisher Scientific, Waltham, MA, USA), following the manufacturer’s instructions. The extracted RNA was reverse-transcribed into cDNA using random primers, and rRNA was subsequently removed. The processed cDNA was fragmented and constructed into a library with an average length of 500 bp. The integrity and concentration of the RNA were assessed using Agilent 2100 Bioanalyzer (Agilent Technologies, Santa Clara, CA, USA) and NanoDrop spectrophotometer (Thermo Fisher Scientific, Waltham, MA, USA). The enriched lncRNA was then used to construct a cDNA library employing a protocol compatible with lncRNA sequencing. The library was subsequently sequenced using an Illumina HiSeq platform. Quality control measures were implemented to filter out low-quality sequences, and bioinformatics analyses were performed on the resulting data to identify and characterize lncRNAs.

### Organelle genome assembly

For plastome assembly, we utilized GetOrganelle v1.7.4.1 with the following parameters: ‘-R 15 -k 21,45,65,85,105 -F embplant_pt’ to assemble the Illumina short-reads (Jin et al., 2020). GetOrganelle generated two complete plastome sequences, and we selected the one where the SSC region aligns in the same direction as *Arabidopsis thaliana* (NC_000932.1). Subsequently, we performed *de novo* assembly for long-reads. The long-reads were polished using Canu (Koren et al., 2017) and then assembled using PMAT assembler (Bi et al., 2024a) with the default parameters. BLASTn (Chen et al., 2015) was employed to identify the draft mitogenome from the assembled sequences. Six mitochondrial contigs were successfully identified. Considering the low accuracy of long-reads from Oxford Nanopore sequencing, we further utilize assembled mitochondrial sequences as the reference sequence. We establish an index using BWA (Li and Durbin, 2009). Subsequently, ‘bwa mem’ was utilized to obtain reads successfully mapped to the reference sequence. Indexing and mapping of the long-reads were performed using minimap2 (Li, 2018), with specific parameters ‘minimap2 -d’ for index creation and ‘minimap2 -ax map-ont -t 8 –secondary=no’ for mapping long-reads. Finally, we performed hybrid assembly using Unicycler (Wick et al., 2017) by combining Illumina short-reads and Nanopore long reads. The mapped Illumina short-reads were initially assembled using SPAdes (Bankevich et al., 2012), and then the Nanopore long-reads were employed to resolve repetitive sequence regions in the assembly, using minimap2 (Li, 2018). After multiple iterations and adjustments, we determined the optimal kmer value of 89. The resulting GFA format files generated by Unicycler were visualized using Bandage (Wick et al., 2015). Ultimately, Unicycler generated a complete circular genome. Notably, as these contigs were assembled based on Illumina short-reads, no additional polishing steps were necessary.

### Verification of the mitogenome structure

In our study, we employed PCR experiments to investigate the structure of *A. odorata*. Specifically, we designed eight specific primers to verify the accuracy of assembly generated by PMAT assembler. The primer design was conducted using the Primer designing tool on NCBI (https://www.ncbi.nlm.nih.gov/tools/primer-blast/) with default parameters. The primer sequences used for PCR reactions are listed in **Supplementary Table 1**. Subsequently, DNA was extracted, and the amplifications were performed using a Pro-Flex PCR system (Applied Biosystems, Waltham, MA, USA). The PCR reaction volume was 25 µL, comprising 2 µL of template DNA, 0.5 µL of forward primer, 0.5 µL of reverse primer, 12.5 µL of 2 × Taq PCR Master Mix, and 9.5 µL of ddH_2_O. The amplification conditions consisted of an initial denaturation at 94 °C for 5 min, followed by 30 cycles of denaturation at 94 °C for 30 s, annealing at 58 °C for 30 s, extension at 72 °C for 60 s, and a final extension step at 72 °C for 5 min. The PCR amplicons were visualized using 1% agarose gel electrophoresis. Subsequently, the single bright bands were excised and sent to Sangon Biotech (Shanghai, China) Co., Ltd. for Sanger sequencing.

### Mitogenome and plastidial genome annotation

The plastome of our *A. odorata* was annotated using CPGAVAS2 (Shi et al., 2019). The plastome of published *A. odorata* (NC_048994.1) was used as the reference genome. The annotation results were further verified using CPGView (Liu et al., 2023) to ensure accurate gene annotations. We utilized IPMGA (http://www.1kmpg.cn/ipmga/) to annotate the assembled mitogenome of *A. odorata*. We selected a database of mitochondrial genes of angiosperms on IPMGA. IPMGA generates annotated files in the standard GenBank format. The tRNA annotations were performed using tRNAscan-SE (Lowe and Eddy, 1997) while rRNA annotations were obtained through BLASTn (Chen et al., 2015). To ensure accuracy, manual edits were made to the annotations using Apollo (Lewis et al., 2002). Finally, the genome map was generated using OGDRAW (v1.3.1) (Alverson et al., 2010).

### Repetitive elements

The long tandem repeats were detected by Tandem Repeats Finder (TRF, https://tandem.bu.edu/trf/trf.html) with the default parameters (Benson, 1999). The simple sequence repeats (SSRs) of the assembled mitogenome were identified using the online website MISA (https://webblast.ipk-gatersleben.de/misa/), the parameters of the minimum numbers of mono-, di-, tri-, tetra-, penta-, and hexanucleotides were set as 10, 5, 4, 3, 3, and 3, respectively. Additionally, forward, reverse, palindromic, and complementary repeat sequences were identified using REPuter (https://bibiserv.cebitec.uni-bielefeld.de/reputer/) with the following settings: hamming distance of three and minimal repeat size of 30 bp, and e-value is limited to less than 1e-5. The visualization of the repetitive elements was done using the Circos package (Zhang et al., 2013).

### Codon usage of mitochondrial genes

We employed PhyloSuite software (v1.2.2) (Zhang et al., 2020a) to parse the GenBank format file of the *A. odorata* mitogenome, extracting the protein-coding genes (PCGs). Subsequently, we conducted an analysis of the codon usage in mitochondrial PCGs using Mega 7.0 software (Kumar et al., 2016), which involved the calculation of Relative Synonymous Codon Usage (RSCU) values. An RSCU value of 1 signifies a neutral preference for codon utilization, whereas an RSCU value exceeding 1 indicates a relatively higher frequency of codon usage.

### Identification of the mitochondrial plastid sequences

To identify the mitochondrial plastid DNAs (MTPTs), we compare the plastome and mitogenome DNAs of *A. odorata* by using BLASTn (Chen et al., 2015) program with the following parameters: -evalue 1e-5, -word_size 9, -gapopen 5, - gapextend 2, -reward 2, -penalty -3. The BLASTn results were visualized using Circos package (Zhang et al., 2013). The identified MTPTs were also annotated by using GeSeq.

### Analysis of RNA editing sites

We employed a two-step approach for predicting RNA editing sites. Initially, lncRNA-Seq reads were mapped to the coding sequences (CDS) of each protein-coding gene (PCG) using BWA software (Li and Durbin, 2009) with default parameters. Subsequently, we utilized REDItools (Picardi and Pesole, 2013) to predict RNA editing sites based on the mapping results. The prediction criteria were established as follows: coverage exceeding 30, frequency equal to or greater than 0.1, and *p*-value equal to or greater than 0.05. Afterward, the Illumina short-reads of DNA were aligned to the CDS of each PCG using BWA software with default parameters. Genomic SNPs were predicted using BCFtools (Li, 2011) based on the mapping outcomes, with thresholds set at coverage greater than 30 and frequency less than or equal to 0.1. These heterogeneous sites of natural variation need to be excluded from RNA editing sites. Finally, after excluding SNP sites, the remaining sites identified in the lncRNA-seq mapping will be considered as genuine RNA editing sites.

To confirm the accuracy of the predicted RNA editing sites, we further designed experiments to validate these four specific sites. The primers were designed on both sides of the editing sites (**Supplementary Table 2**), and amplification was performed using genomic DNA (gDNA) and cDNA obtained from RNA reverse transcription using random primers as templates. The reaction conditions for amplification are described above. The amplified products were subsequently subjected to Sanger sequencing. Finally, by comparing the sequences of the products obtained from gDNA and cDNA, we determined the occurrence of RNA editing events.

### Collinear analysis

For the collinear analysis with *A. odorata*, we selected five closely related species: *Citrus unshiu* (NC_057142.1), *Citrus maxima* (NC_057143.1), *Citrus sinensis* (NC_037463.1), *Toona ciliata* (NC_065060.1) and *Toona sinensis* (NC_065061.1). We identified collinear blocks based on sequence similarity using the BLASTn program with the following parameters: -evalue 1e-5, -word_size 9, -gapopen 5, - gapextend 2, -reward 2, -penalty -3. Only collinear blocks longer than 1 kb were retained for downstream analysis. To visualize the collinear relationships, we generated a multiple synteny plot using TBtools (Chen et al., 2023).

### Phylogenetic analysis

We retrieved a total of 31 mitogenomes, including two outgroups (*Stylosanthes capitata* and *Glycine max*), from the GenBank database. These mitogenomes were used to construct a phylogenetic tree with *A. odorata*. Firstly, PhyloSuite (v.1.2.2) (Zhang et al., 2020a) was employed to identify and extract orthologous protein-coding genes (PCGs) across the analyzed species. The nucleotide sequences corresponding to these PCGs were then aligned using MAFFT (v7.471) (Katoh and Standley, 2013). Subsequently, the aligned sequences were concatenated to generate the input for phylogenetic tree construction. The maximum likelihood (ML) method was implemented using IQ-TREE (version 2.1.4-beta) (Minh et al., 2020) with the parameters “–alrt 1000 -B 1000”. Using the Bayesian Information Criterion (BIC) for model selection, the results indicate that the best-fit model is GTR+F+R2. The bootstrap analysis was performed with 1,000 replicates. Finally, the resulting phylogenetic tree was visualized and edited using the online tool ITOL (Letunic and Bork, 2019).

## Results

### Genomic structure of the *A. odorata* mitogenome

**Figure 1:**
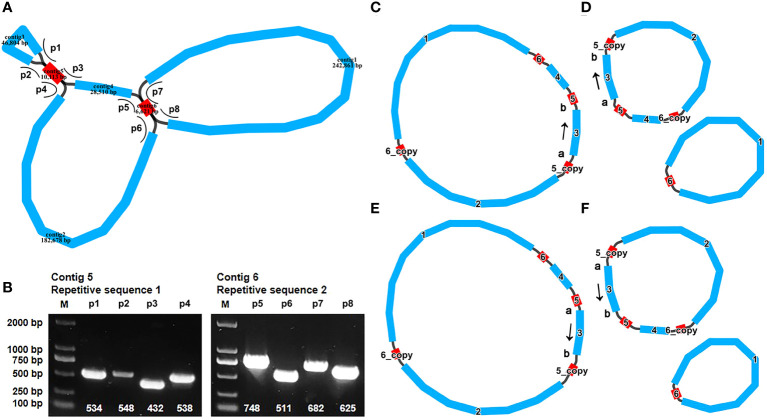
The graphic assembly and verification of *A odorata* mitogenome. **(A)**. The graphic mitogenome consists of six contigs with different lengths, and they connected to each other. The length of the six contigs are 242,861 bp, 182,678 bp, 46,804 bp, 28,510 bp, 10,113 bp and 6,621 bp, respectively. Contig 5 and contig6 are the repetitive sequence. **(B)**. represents the electropherogram of eight paths. **(C-F)** represent four conformations mediated by two pairs of repeat sequences, respectively. The arrows in panel **(C-F)** indicates the direction of sequence 3. These four conformations can dynamically change between them.

The assembly is composed of six distinct nodes and eight edges (paths), visually depicted in **Figure 1A**. Each of these nodes signifies an assembled contig, demonstrating a region of overlap along the linkages. It’s worth noting that contig5 and contig6 exhibited distinct characteristics suggestive of potential repetitive sequences. These two repeat sequences each showcased four distinct paths (designated as p1–p4 and p5–p8, **Figure 1B**). To confirm the presence of these paths within the *A. odorata* mitogenome, we conducted PCR experiments. The four primer pairs (F1 + R1, F2 + R1, F3 + R3, F3 + R2) were employed to validate the repeated sequence within contig5 and the paths, while the remaining pairs (F4 + R4, F5 + R5, F6 + R4, F7 + R7) were utilized to confirm the repetitive sequence within contig6 and the paths. The PCR products exhibited conformity with the anticipated outcome (**Supplementary Figure 1**). The results of these PCR experiment not only validated the accuracy of the assembly and the eight paths, but also helped us to propose four possible genomic configurations. Configuration 1 presents a master circular structure, incorporating all six contigs (**Figure 1C**). In configuration 2, contig2, contig3, contig4, contig5, and contig6 collectively form a circular arrangement, and contig1 and contig6 form a smaller circular (**Figure 1D**). Similarly, configuration 3 showcases a circular arrangement encompassing all six contigs, while the contig3 was inverted compare to configuration 1 (**Figure 1E**). Lastly, configuration 4 is similar to configuration 2, with the inversion of contig3 (**Figure 1F**). Our PCR experiments show the possibility of multiple configurations, and here we use configuration 1, a master circle represent the complete mitogenome, for subsequent analysis.

### Gene content of the *A. odorata* mitogenome

**Figure 2:**
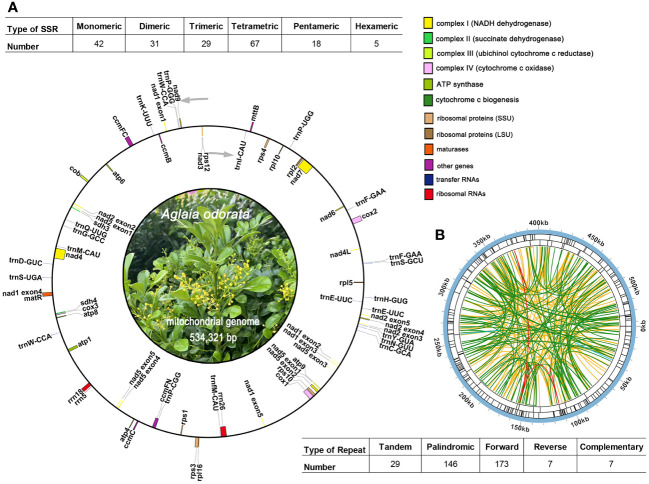
The mitogenome map of *A odorata*. **(A)**. The figure shows the master circle of *A odorata* mitogenome. Genes transcript clockwise or counter-clockwise strands are drawn on the upper or lower of the circles, respectively. Genes belonging to different functional groups are color-coded. The table showing the number of SSRs of each type, with tetrameric being the most and Hexametric being the least. **(B)**. The identified dispersed repeats (≥ 30bp). The green ribbons represent the forward repeats, the yellow ribbons represent the palindromic repeats, the red ribbons represent the complementary repeats and the blue ribbons represent the reverse repeats. The detailed information about dispersed repeats can be found in **Table S6**.

The mitogenome maps of *A. odorata* was visually presents in **Figure 2A**. The total length of *A. odorata* mitogenome is 534,321 bp, and consists of 35 distinct protein-coding genes (PCGs) (**Table 1**), including five ATP synthase genes (*atp1, atp4, atp6, atp8, and atp9*), four cytochrome c biogenesis genes (*ccmB, ccmC, ccmFC*, and *ccmFN*), nine NADH dehydrogenase genes (*nad1, nad2, nad3, nad4, nad4L, nad5, nad6, nad7*, and *nad9*), three cytochrome c oxidase genes (*cox1, cox2*, and *cox3*), one transport membrane protein gene (*mttB*), one maturases gene (*matR*), and one cytochrome b gene (*cob*), four large subunits of ribosomal proteins (*rpl2, rpl5, rpl10*, and *rpl16*), five small subunits of ribosomal proteins (*rps1, rps3, rps4, rps10* and *rps12*), as well as two succinate dehydrogenases (*sdh3* and *sdh4*).

**Table 1:** Gene composition in the mitogenome of *A. odorata*.

Group of genes	Name of genes
ATP synthase	*atp*1, *atp*4, *atp*6, *atp*8, *atp*9
NADH dehydrogenase	*nad*1, *nad*2, *nad*3, *nad*4, *nad*4L, *nad*5, *nad*6, *nad*7, *nad*9
Cytochrome *b*	*cob*
Cytochrome *c* biogenesis	*ccmB, ccmC, ccmFC, ccmFN*
Cytochrome *c* oxidase	*cox*1, *cox*2, *cox*3
Maturases	*mat*R
Transport membrane protein	*mtt*B
Succinate dehydrogenase	*sdh*3*, sdh*4
Ribosomal protein large subunit	*rpl2, rpl*5, *rpl*10, *rpl*16
Ribosomal protein small subunit	*rps*1, *rps*3, *rps*4, *rps*10, *rps*12
Ribosome RNA	*rrn5*, *rrn18*, *rrn26*
Transfer RNA	*trnS-GCU, trnF-GAA* (×2)*, trnP-UGG, trnI-CAU, trnP-GGG, trnW-CCA* (×2)*, trnK-UUU, trnQ-UUG, trnG-GCC, trnM-CAU, trnD-GUC, trnS-UGA, trnP-CGG, trnfM-CAU, trnC-GCA, trnN-GUU, trnY-GUA, trnE-UUC* (×2)*, trnH-GUG*

In the *A. odorata* mitogenome, a total of 22 tRNA genes have been annotated, with 19 being unique. Among these, 11 tRNA genes are mitochondrial native. Furthermore, our investigation has revealed 7 tRNA genes originating from the plastid: *trnN-GUU, trnH-GUG, trnM-CAU, trnD-GUC, trnW-CCA, trnP-UGG*, and *trnI-CAU*. Notably, our exploration has led us to the identification of a tRNA gene with bacterial origins, *trnC-GCA*, exhibiting a remarkable level of sequence homology with previously documented genes (Kitazaki et al., 2011). The remaining tRNA genes, devoid of sequence homology with known organelle tRNA genes, are unknown about their origin (Li, 2011; Rice et al., 2013). Furthermore, we have successfully pinpointed three distinct rRNA genes within the *A. odorata* mitogenome, namely *rrn5, rrn18*, and *rrn26*. The precise positions of each gene can be referenced in **Supplementary Table 3**. Among the entirety of the genes that have been annotated, 10 PCGs encompass introns (**Supplementary Table 3**). To elaborate, the genes *ccmFC*, *cox2, cox1*, *trnP-CGG*, and *rps10* each feature one intron, whereas *nad4* encompasses two introns. On the other hand, the genes *nad1, nad2, nad5*, and *nad7* encompass four introns each.

### Repetitive elements

Microsatellites, also known as simple sequence repeats (SSRs), typically consist of tandem sequences with a length of up to 6 base pairs in eukaryotic genomes. Within the mitogenome of *A. odorata*, a comprehensive total of 192 SSRs has been meticulously identified (as shown in **Supplementary Table 4**). Among this array of SSRs, tetrameric repeats stand out as the most prominent, encompassing 34.90% (67) of the overall count. This pattern is subsequently trailed by monomeric repeats (42), dimeric repeats (31), trimeric repeats (29), pentameric repeats (18), and hexametric repeats (5) (**Figure 2A**). And we have detected 29 long tandem repeat elements (**Supplementary Table 5**).

In addition, we have meticulously identified a total of 333 pairs of dispersed repeats within the *A. odorata* mitogenome, each with lengths equal to or exceeding 30 base pairs. This collection encompasses 173 pairs of forward repeats, 146 pairs of palindromic repeats, and 7 pairs of reverse repeats and complementary repeats, as outlined in **Supplementary Table 6**. Most of these repeat elements exhibit a length of less than 200 bp, and it’s evident that the number of dispersed repeats surpasses that of both SSRs and tandem repeats. The only two long dispersed repeats identified in the *A. odorata* mitogenome is contig5 and contig6 (10,113 bp palindromic repeat element and 6,621 bp repeat element). Cumulatively, the extent of these dispersed repeats encompasses 30,754 bp, constituting 5.76% of the entire *A. odorata* mitogenome. Furthermore, we provide a visual representation employing the Circos (v1.120) package (Krzywinski et al., 2009) to depict the dispersed repeats of the *A. odorata* mitogenome and counted the number of repetitive sequences in **Figure 2B**. These dispersed repeats are distributed in various regions of the mitogenome, effectively increasing the size of the genome.

### Codon usage analysis

**Figure 3:**
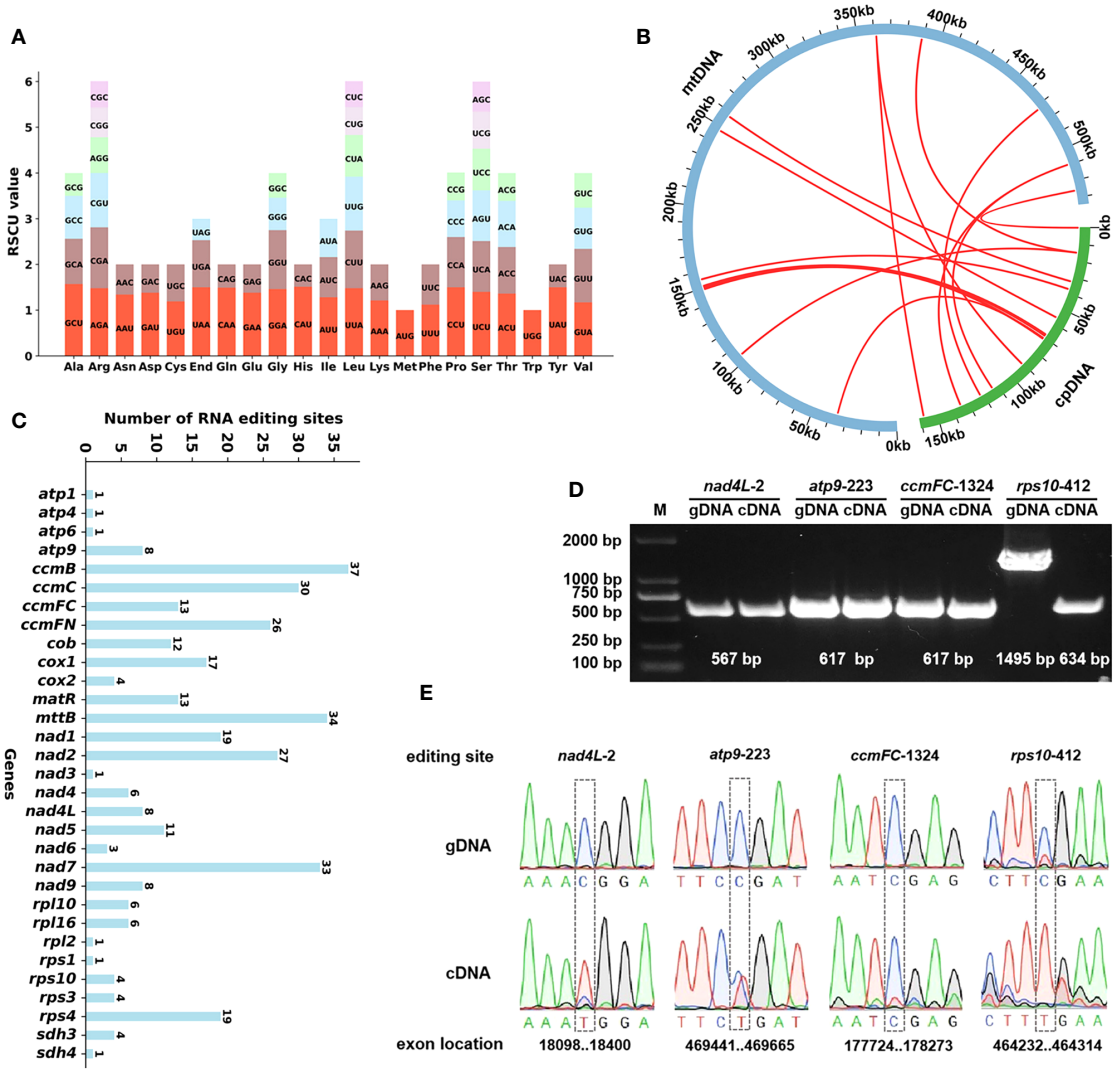
**(A)** The codon usage and RSCU value of *A odorata* PCGs. Codon families are shown on the x-axis. RSCU values are the number of times a particular codon is observed relative to the number of times that codon would be expected for uniform synonymous codon usage. **(B)** Schematic representation of the distribution of MTPTs between the mitogenome and the plastome of *A odorata*. The MTPTs on the chloroplast IR regions were counted only once. The location of each MTPT has been marked on the Figure. **(C)** Characteristics of the RNA editing sites identified in mitochondrial PCGs of *A odorata.* The ordinate shows the number of RNA editing sites identified in PCGs, the abscissa shows the name of PCGs identified in the mitogenome of *A odorata*. **(D)** The figure shows the results of PCR experiments on cDNA and gDNA of four genes. The experimental results are as expected. gDNA amplification length of *rpl10-412* gene is longer because of the presence of intron in the gene. **(E)**. The figure shows the results of Sanger sequencing experiments on cDNA and gDNA of four genes, and it shows that RNA editing is not universally present in all cases.

We conducted an analysis of the codon usage within the PCGs. The comprehensive codon usage data for all PCGs is presented in **Supplementary Table 7**. As shown in **Figure 3A**, revealing a discernible preference for specific codons among mitochondrial protein-coding genes. Notably, the RSCU values for the start codons AUG (Met) and UGG (Trp) both equate to 1. Furthermore, the RSCU values for the termination codons UGA (End), UAA (End), and UAG (End) are recorded as 1.20, 1.02, and 0.78, respectively. In terms of specific codons, GCU (Ala), UAA (End), CAU (His), CCU (Pro), and UAU (Tyr) emerge as the four most frequently employed codons within *A. odorata*. Conversely, GCG (Ala), UAG (End), CAC (His), and UAC (Tyr) are identified as the four least utilized codons. A visual representation in **Figure 3A** underscores the prevalence of arginine (Arg), leucine (Leu), and serine (Ser) codons, while methionine (Met) and tryptophan (Trp) codons exhibit relatively lower occurrence rates.

### Characteristic of mitochondrial plastid DNAs

In our study, we conducted an annotation of the plastidial genome of *A. odorata* and performed a comprehensive comparison with its mitogenome. Employing the BLASTn program, we successfully identified a total of 12 instances of homologous sequences, we considered they might be potential MTPTs occurring between these two organelle genomes. These 12 MTPTs collectively span a length of 2,501 bp, contributing to 0.47% of the mitogenome’s total size (**Supplementary Table 8**). Among these MTPTs, MTPT12 stands out as the longest, spanning 1,122 bp, while MTPT1 emerges as the briefest, encompassing a mere 29 bp. Subsequently, our efforts focused on annotating these MTPTs, revealing a revelation: every MTPT encompassed plastidial genes or gene fragments. As depicted in **Figure 3B** and **Supplementary Table 8**, MTPT12 harbors a set of plastid genes, primarily associated with the photosystem II protein complex. It included *psbJ, psbL, psbF*, and *psbE*. Moreover, our analysis unearthed various gene fragments that resulted from the process of plastid migration. These fragments encompassed genes such as *petG, ndhD, psbC*, and *atpH*. It is plausible that these gene fragments underwent sequence loss during migration.

### Analysis of RNA editing sites

We have successfully discerned a total of 427 high-confidence C to U RNA editing sites across 32 mitochondrial protein-coding genes (**Supplementary Table 9**). These editing sites are supported by our lncRNA data with an average depth of nearly 5100 times. The RNA editing sites for each gene are visually represented in **Figure 3C**. Within this set of mitochondrial genes, *ccmB* boasts the highest number of RNA editing sites at 45, closely followed by *mttB* with 41, positioning them as the foremost two genes in terms of RNA editing occurrences. Conversely, genes like *atp1, atp6* and *rpl5* possess the least number of editing sites, with only a solitary C to U edit detected for each. Based on our findings, we have identified C to U RNA editing events in three genes, which lead to the creation of premature stop codons. These genes are *ccmFC, atp9* and *rps10* (where CGA transitions to UGA). Notably, RNA editing plays a role in the formation of start codons as well, such as gene *nad4L*, have their start codons generated through RNA editing, converting ACG codon to AUG.

To evaluate the precision of this prediction, we employed PCR amplification and Sanger sequencing as a means of substantiating the occurrence of RNA editing. The four primer sequences can be found in **Supplementary Table 2**. Among these, every RNA site underwent successful verification, namely *nad4L*-2, *atp*9-223, *ccmFC*-1324, and *rps*10-412 (as depicted in **Figure 3D**). And all the validated sites encompassed C to U substitutions (**Figure 3E**), *ccmFC*-1324 is an exception, probably because of the lower frequency of editing, but the peak plot shows a hybrid peak of base U (uracil). Notably, the genomic DNA (gDNA) of *rps10* has a longer PCR product because our primers span its introns, and the banding here also confirms that the DNA was sufficiently removed in the cDNA experimental group. Moreover, it’s noteworthy that each of these sites involved non-synonymous substitutions, as detailed in **Supplementary Table 9**. These PCR experiment firmly attesting to the dependability of the anticipated RNA editing sites.

### Collinear and phylogenetic analysis

**Figure 4:**
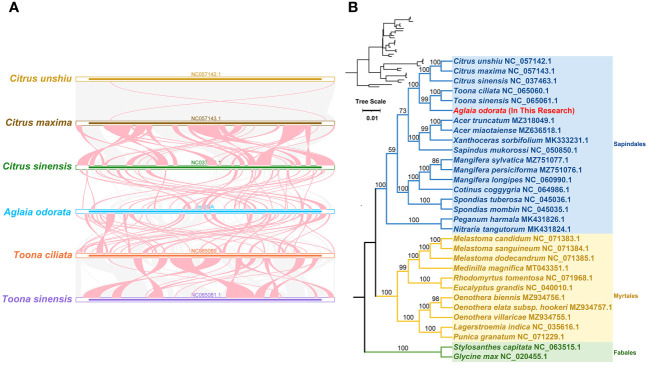
**(A)** Collinear analysis of *A odorata* mitogenome and its related species. The colorful bars indicated the mitogenomes, and the ribbons showed the homologous sequences between the adjacent species. The blue ribbons indicate regions with homology and the red ribbons indicate where the inversion occurred. The homologous blocks less than 0.5 kb in length are not remaining, and regions that fail to have a homologous block indicate that they are unique to the species. **(B)** The phylogenetic relationships of *A odorata* and another 30 species based on conserved mitochondrial genes. The tree was constructed based on the nucleotide sequences of conserved mitochondrial protein-coding genes (PCGs). We used Maximum Likelihood (ML) method to reconstruct the phylogenetic tree. The ML topology is indicated with ML bootstrap support values. *T. capitata* and *G max* were used as outgroups. The species list and its accession numbers that used in phylogenetic analysis are shown in **Table S10**.

To delve into the rearrangements and conserved sequence blocks within the mitogenomes, we utilized the BLASTn program to pinpoint homologous collinear blocks. Illustrated in **Figure 4A**, each ribbon connecting two neighboring mitogenomes signifies a remarkably homologous collinear block or sequence. When comparing *A. odorata* and *T. ciliata*, we observed one large adjacent collinear block. Furthermore, we uncovered three collinear blocks exceeding 10 kb in length between these two mitogenomes. However, in the case of comparing *A. odorata* with *C. sinensis*, no extensive collinear blocks were detected, with the longest identified block measuring only 7.5 kb. Overall, while longer collinear blocks tend to be present between closely related species, the mitogenomes demonstrated limited collinearity, with several regions lacking homology. These findings underscore the prevalence of extensive genomic rearrangements between *A. odorata* and its related mitogenomes, suggesting that the genomic structure of the mitogenomes is not conserved.

In addition, we carried out a phylogenetic analysis utilizing 31 mitogenomes of related species, with *T. capitata* and *G. max* serving as outgroups for reference. The detailed list of species and their corresponding GenBank accessions utilized for this analysis is available in **Supplementary Table 10**. By aligning and concatenating the shared protein-coding genes (PCGs), we created the matrix data for analysis. The outcome of our phylogenetic investigation yielded a maximum likelihood (ML) tree that exhibits robust support along the primary basal branches (refer to **Figure 4B**). In terms of evolutionary relationships, *A. odorata* exhibits its closest affinity with *T. ciliata*, both species falling under the Sapindales order. While the overall structure of the phylogenetic tree broadly aligns with the APG IV system (The Angiosperm Phylogeny et al., 2016) at the order level, it’s noteworthy that within the Sapindales order, a couple of nodes lack bootstrap support. This suggests that deriving phylogenetic inferences solely from mitochondrial PCGs might have limitations in accurately resolving lower taxonomic categories.

## Discussion

Our study successfully assembled the complete mitogenome of *A. odorata*, with a total length of 534,321 bp. To accomplish this task, we employed a hybrid assembly approach, combining both Illumina short-reads and Oxford Nanopore long-reads. In contrast to the stability observed in plant plastomes, plant mitogenomes have undergone significant transformations throughout evolution, resulting in complex structures (Bi et al., 2022; Han et al., 2022; Ma et al., 2022; Bi et al., 2024b). Numerous researchers have delved into the intricate structural variations within plant mitogenomes (Chevigny et al., 2020), leading to the development of various tools for decoding these dynamically evolving genomes (He et al., 2023; Shan et al., 2023). Some studies propose that the diversity within mitogenomes may arise from long repeat-mediated recombination (Wang et al., 2023a). Within the mitogenome of *A. odorata*, our analysis unveiled two pairs of lengthy repetitive sequences, measuring 10,113 bp and 6,621 bp, respectively. This prolific presence of repeats points towards their potential significance not only in genome reconfiguration but also in influencing genome size dynamics. Remarkably, these two pairs of long repetitive sequences each possibly facilitated two conformations, and up to four potential configurations can be created. This highlights that the mitogenome structure of *A. odorata* is not static but rather dynamically varies among these four conformations. The phenomenon of long repeat-mediated recombination is not unique to *A. odorata*; it has also been observed in other plant species such as *Mimulus guttatus* (Mower et al., 2012)*, Scutellaria tsinyunensis* (Li et al., 2021)*, Photinia serratifolia* (Wang et al., 2023a), and *Ginkgo biloba* (Guo et al., 2016). Furthermore, extensive research has demonstrated that short dispersed repeats contribute to mitogenome recombination in various plant species, including *Nymphaea colorata* (Dong et al., 2018)*, Silene latifolia* (Sloan et al., 2012), and *Ginkgo biloba* (Guo et al., 2016).

Horizontal gene transfer (HGT) among organellar genomes and the nuclear genome is a common phenomenon that plays a pivotal role in plant evolution (Sprinzl and Vassilenko, 2005). The genome structure and evolutionary dynamics of plant mitogenomes render them particularly prone to acquiring and assimilating foreign DNA (Christensen, 2013). In contrast to plastid DNA, plant mitogenomes exhibit a greater propensity to accept and incorporate foreign genetic material, a phenomenon frequently observed. In the case of *A. odorata*, we have observed some sequences transferred from the plastidial genome to the mitogenome (**Supplementary Table 8**, **Figure 3B**). These MTPTs are believed to hold substantial implications for eukaryotic evolution, fostering genetic diversity. Among the MTPTs identified in the *A. odorata* mitogenome, MTPT12 stands out as the longest, spanning 1,122 base pairs. However, in comparison to the mitogenomes of other published species, *A. odorata* displays fewer MTPTs, and they tend to be of shorter lengths. For instance, in *Suaeda glauca*, MTPTs covering 26.87 kb constitute 5.18% of its mitogenome. Some studies have unveiled a notable degree of diversity in MTPTs among various species. Previous research has revealed that tRNAs within plant mitochondria have diverse origins. A portion of these tRNAs is inherited from the ancestral mitochondria, while another part is acquired from chloroplasts through HGT (Sprinzl and Vassilenko, 2005). By leveraging sequence similarities and existing findings, we successfully identified specific tRNA genes in the *A. odorata* mitogenome that originated from the plastid and were transferred to the mitochondria (Richardson et al., 2013). Within the *A. odorata* mitogenome, we identified the following tRNA genes as potential acquisitions from the plastid: *trnN-GUU, trnH-GUG, trnM-CAU, trnD-GUC, trnW-CCA, trnP-UGG*, and *trnI-CAU*. Over the course of evolutionary timescales, these MTPTs have led to the incorporation of functional tRNAs, as evidenced by their widespread conservation across angiosperms (Chaw et al., 2008). For instance, *trnH-GUG* and *trnM-CAU* were early additions to the mitogenome and remain functional (Joyce and Gray, 1989). However, during the transfer of DNA fragments from chloroplasts to the mitogenome, some PCGs are often carried along and tend to become nonfunctional pseudogenes. This phenomenon also occurs within the mitogenome of *A. odorata* (**Supplementary Table Table 8**).

Plant mitochondrial RNA editing is a biological phenomenon where specific nucleotide positions within the mitochondrial RNA sequence undergo base mutations catalyzed by mitochondrial RNA editing enzymes (Wang et al., 2019; Liu et al., 2020; Wang et al., 2023b). These RNA editing processes can convert C to U or U to C in the RNA sequence (Gerke et al., 2020), playing a pivotal role in mitochondrial gene expression and function (Sosso et al., 2012). This is because many of these RNA editing events can result in changes in RNA sequences, leading to variations in the final protein products (Edera et al., 2018). And RNA editing of mitochondrial genes is believed to be an important factor in regulating plant cytoplasmic inheritance-related traits (Liu et al., 2013). In our predictions, we observed that most RNA editing sites occur at the first or second positions of the triplet codon, a pattern similar to that seen in many other plants (Grewe et al., 2014; Kovar et al., 2018). Furthermore, as depicted in **Figure 3E**, the frequency of editing varies greatly at different sites. For example, in gene *ccmFC*-1324, the frequency of RNA editing is lower than that of non-editing events. Furthermore, in our RNA editing experiments, we have successfully confirmed the occurrence of RNA editing events that generate stop codons in gene *ccmFC*, *rps10* and *atp9*. The emergence of RNA editing events that yielded start codons also been found in gene *nad4L*. This new start and stop codons are typically generated to encode proteins that exhibit greater conservation and homology with corresponding proteins in other species, thereby enhancing gene expression within the mitochondria. In future cases, the annotation of these genes should fully account for the influence of RNA editing events, otherwise the wrong coding sequence will be obtained.

## Conclusion

In our study, we have accomplished the successful assembly of the mitogenome of *A. odorata*, revealing a circular genome structure. We conducted thorough analyses to explore its gene content, repetitive elements, codon usage, MTPTs, and RNA editing sites, along with making phylogenetic inferences. To the best of our knowledge, this represents the first comprehensive description of a complete mitogenome within *A. odorata*. Our findings illuminate previously uncharted aspects of the evolutionary dynamics of mitochondrial genes, providing valuable insights into the evolutionary history of mitogenomes.

## Data availability statement

The mitogenome sequence is available in nucleotide database of GenBank (https://www.ncbi.nlm.nih.gov/nucleotide/) with accession numbers: OR680716.1 (plastome) and OR680718.1 (mitogenome). The sequencing reads used for mitogenome assembly in this study have been released on the NCBI with those accession numbers: PRJNA1031331 (BioProject); SAMN37933744 (BioSample) and SRR26513849, SRR26513847 and SRR26513848 (SRA).

## Author contributions

ZH: Data curation, Formal analysis, Funding acquisition, Methodology, Software, Writing – original draft, Writing – review & editing. ZZ: Data curation, Formal analysis, Methodology, Writing – review & editing. JZ: Investigation, Resources, Software, Writing – original draft. XC: Methodology, Resources, Software, Writing – original draft. JL: Supervision, Validation, Writing – review & editing. LL: Investigation, Supervision, Writing – review & editing. YL: Formal analysis, Funding acquisition, Validation, Visualization, Writing – original draft, Writing – review & editing.
