# Supplementary material for: The complete mitochondrial genome of Aglaia odorata, insights into its genomic structure and RNA editing sites
Source: Front Plant Sci. 2024 Mar 6;15:1362045. doi: 10.3389/fpls.2024.1362045 (PMC10950942; doi:10.3389/fpls.2024.1362045)

# Data availability

The mitogenome sequence is available in nucleotide database of GenBank (https://www.ncbi.nlm.nih.gov/nucleotide/) with accession numbers: OR680716.1 (plastome) and OR680718.1 (mitogenome). The sequencing reads used for mitogenome assembly in this study have been released on the NCBI with those accession numbers: PRJNA1031331 (BioProject); SAMN37933744 (BioSample) and SRR26513849, SRR26513847 and SRR26513848 (SRA).

**Figure S1 The Sanger sequencing results of eight paths in the mitogenome of *A. odorata.***

**Figure S1A-H** represent the Sanger sequencing results for each of the eight paths, The sequencing results are expected and verify the reality of these eight paths.

**A**


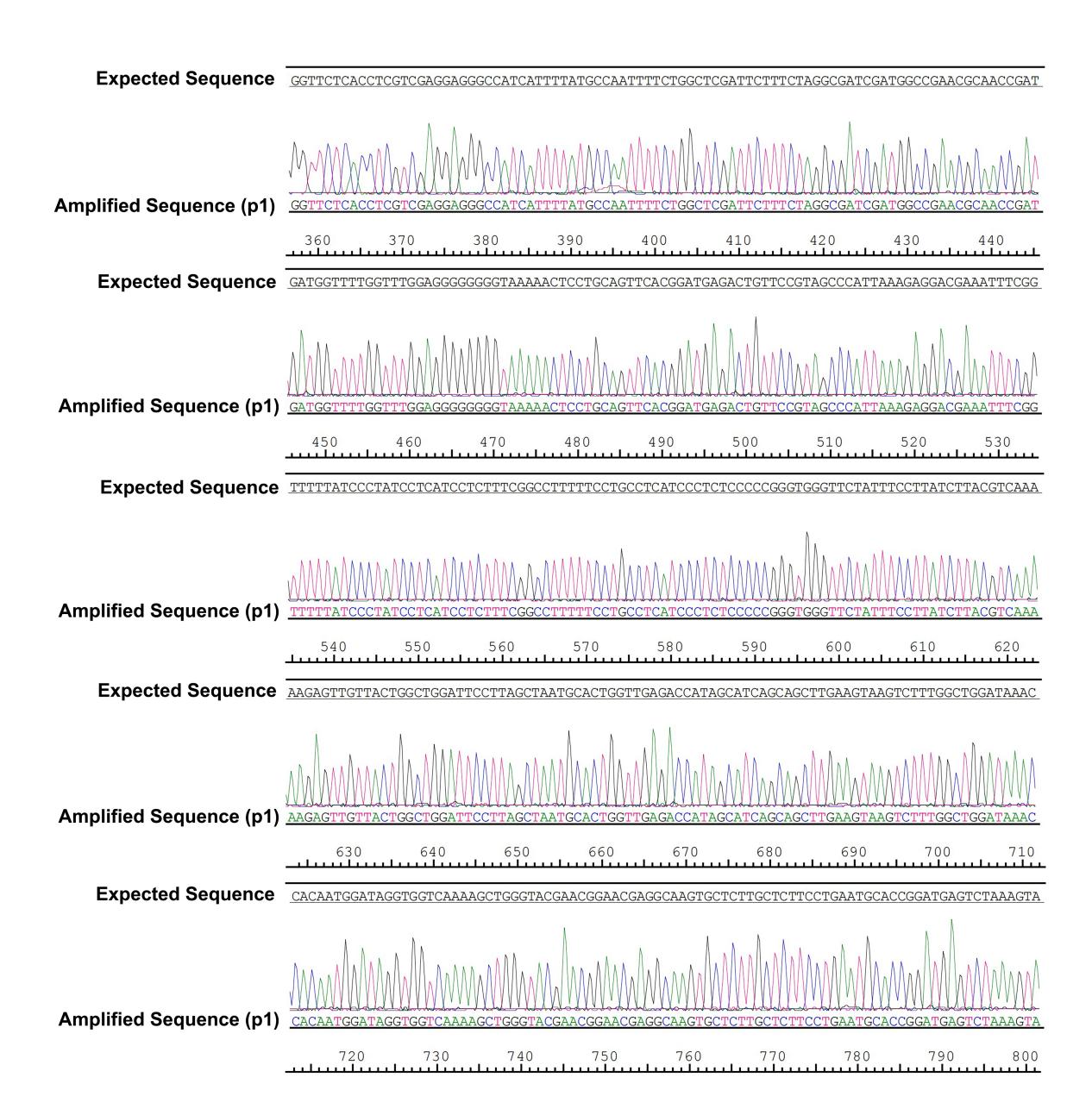


**B**


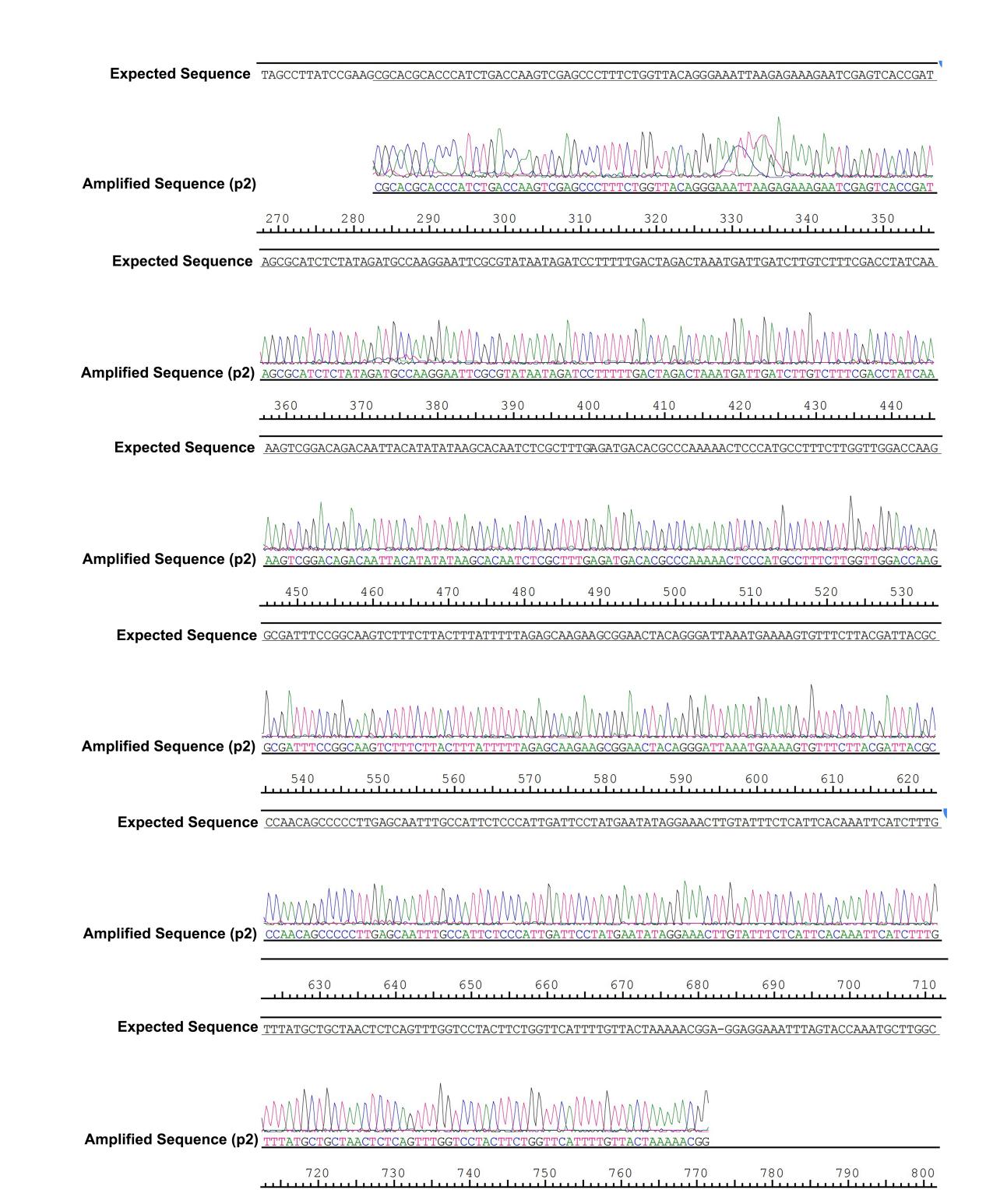


**C**
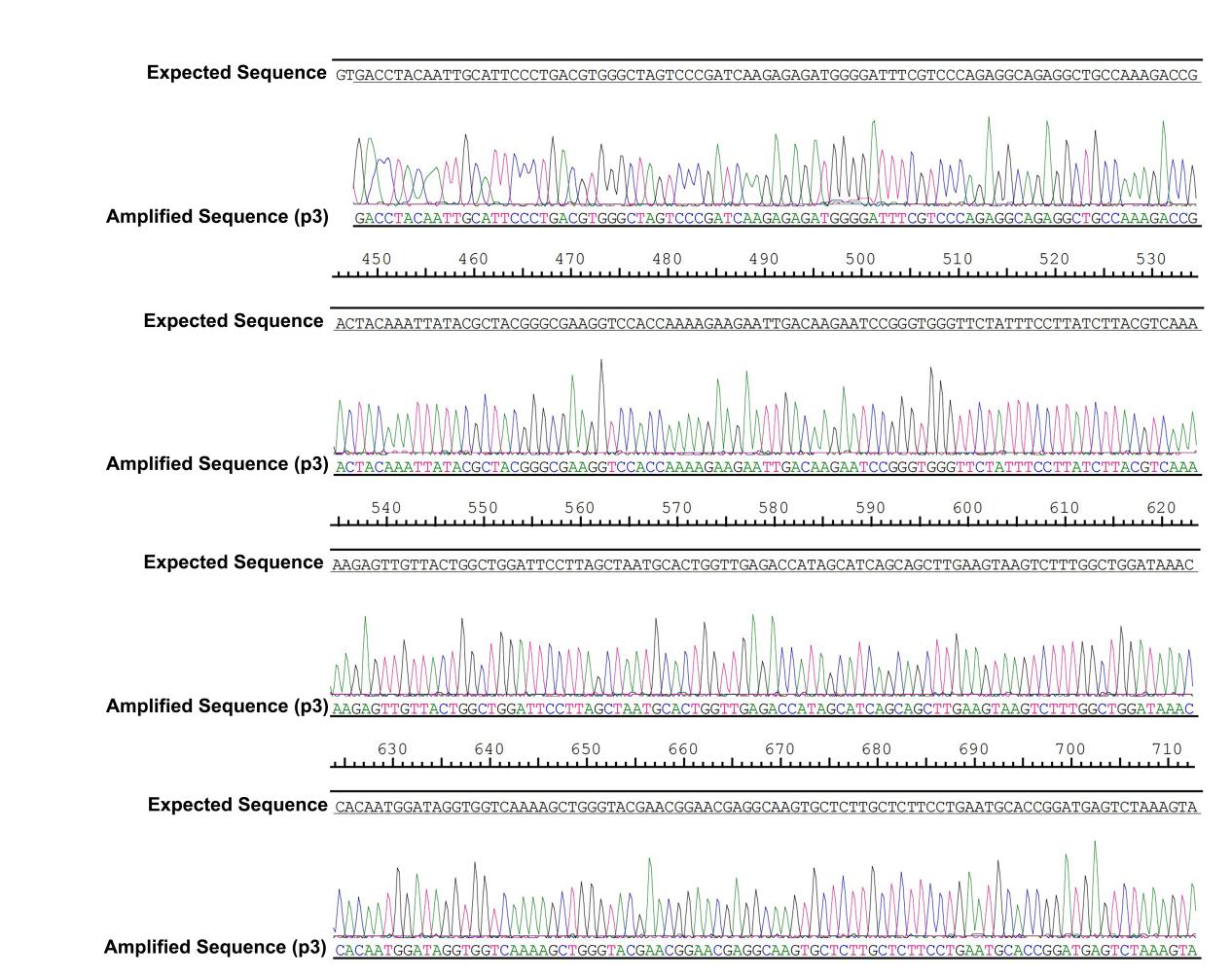


**D**


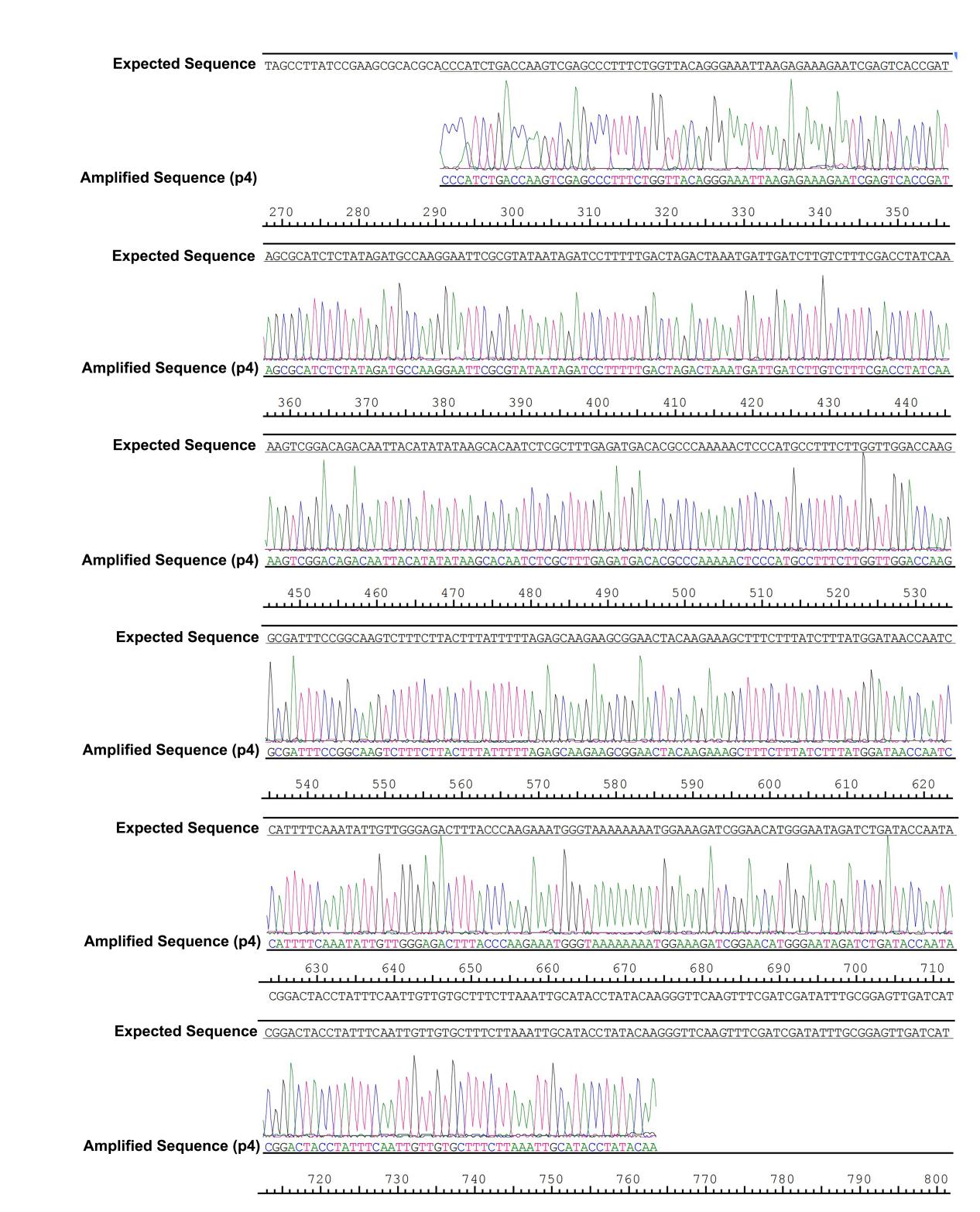


**E**


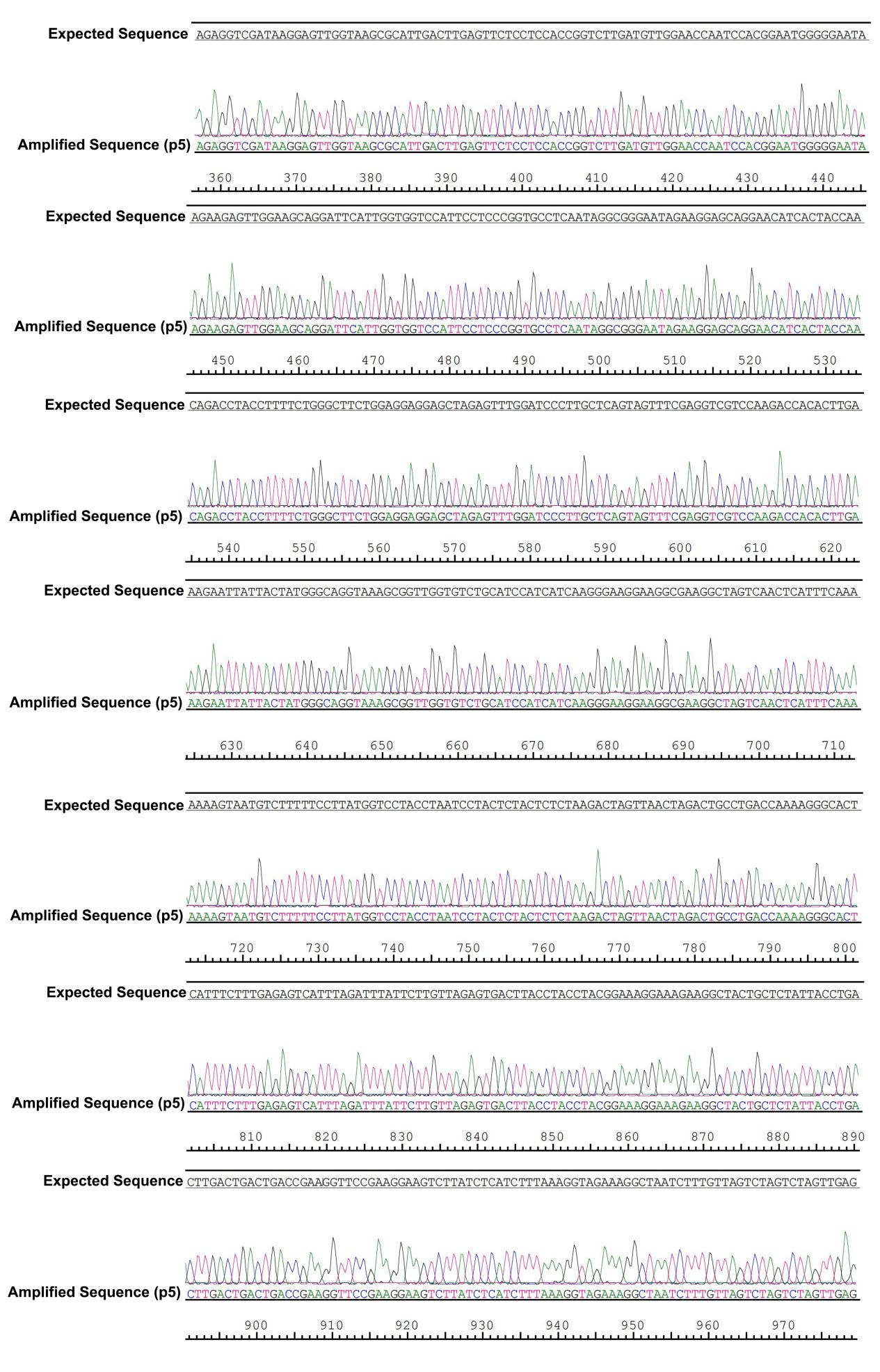


**F**


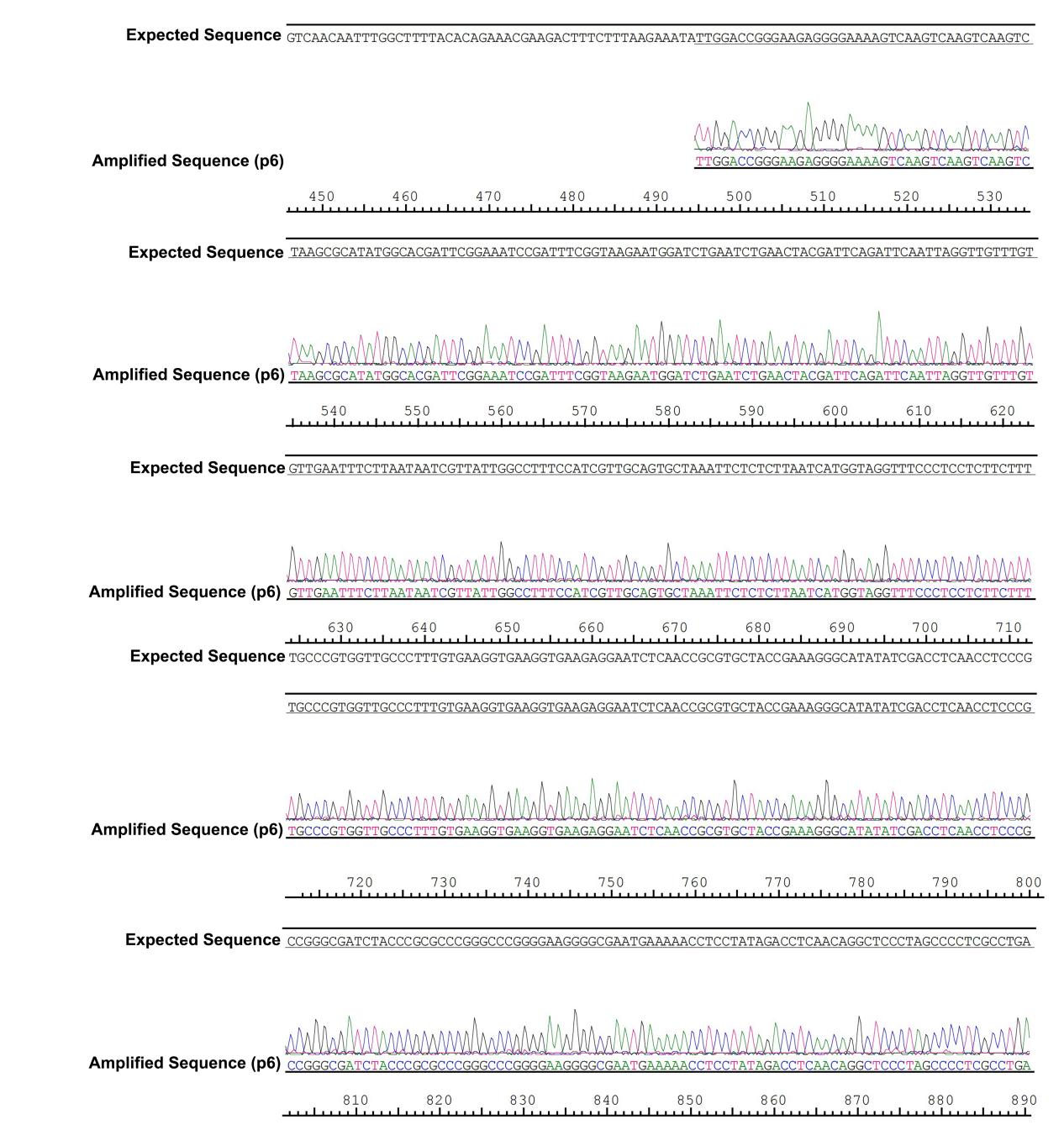


**G**


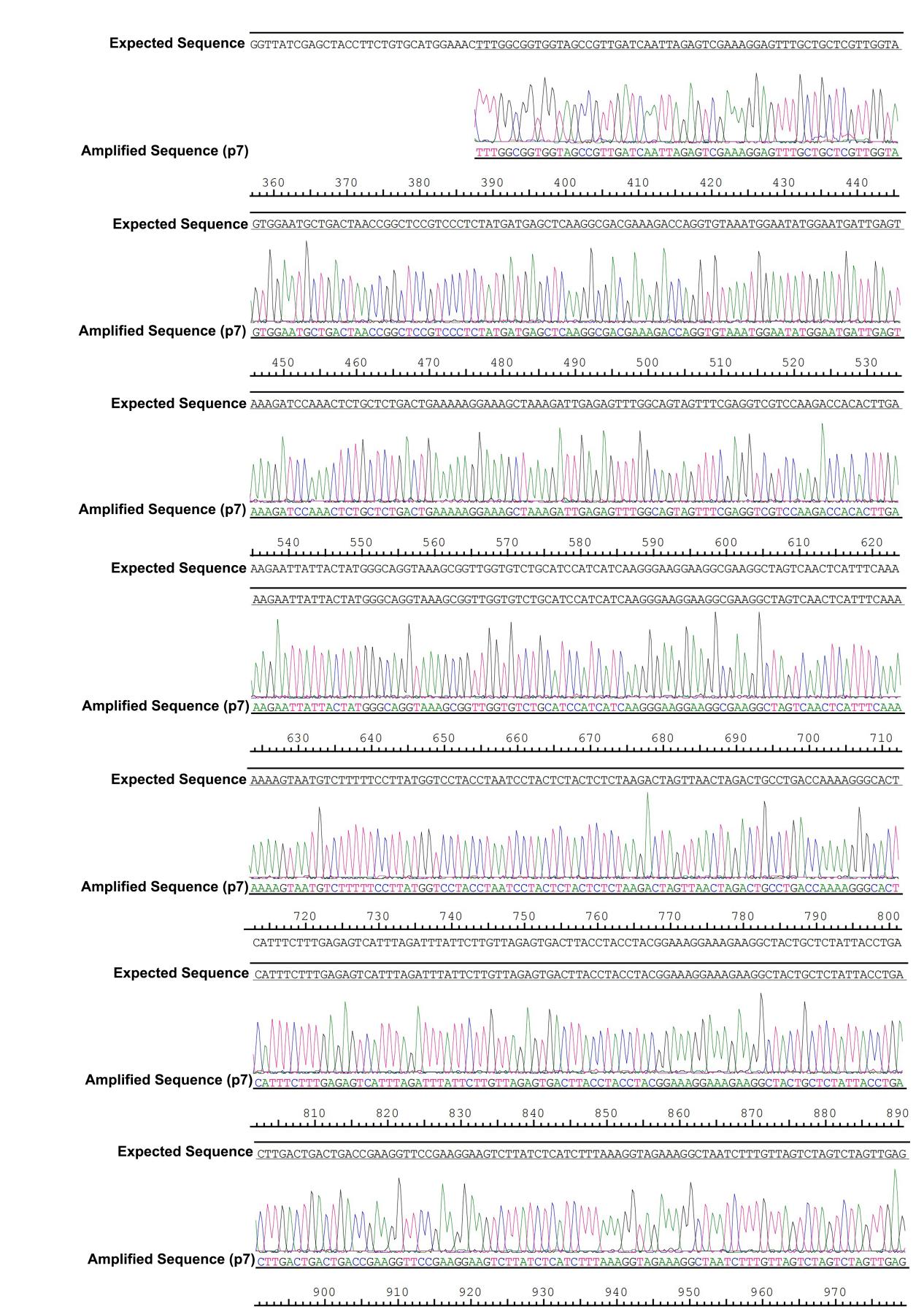


**H**


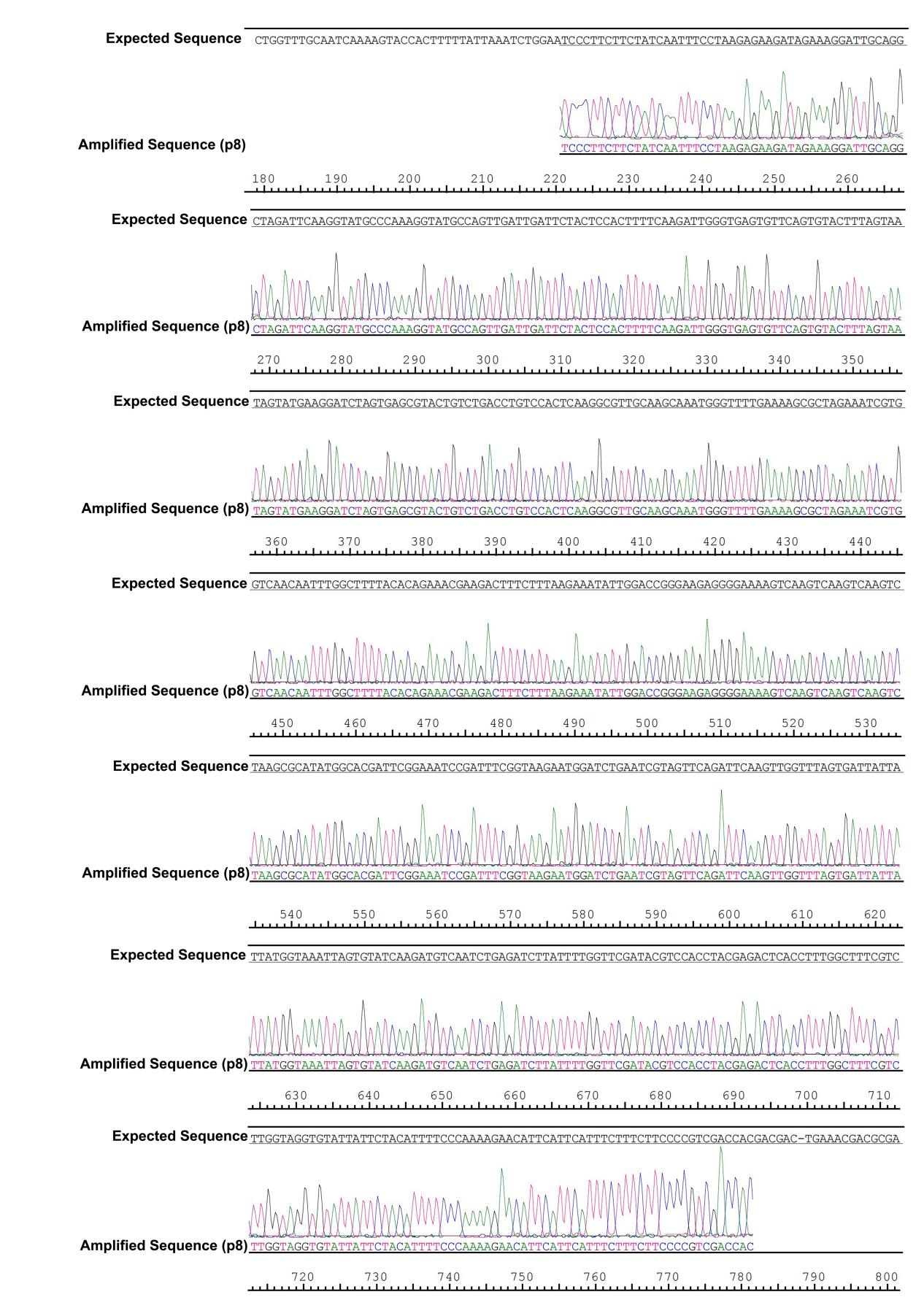

Supplement: Supplementary file 1 [file DataSheet_1.docx]
